# Probiotic Administration Modulates Gut Microbiota and Suppresses Tumor Growth in Murine Models of Colorectal Cancer

**DOI:** 10.3390/ijms26094404

**Published:** 2025-05-06

**Authors:** Anna Niechcial, Marlene Schwarzfischer, Paulina Wawrzyniak, Madita Determann, Doris Pöhlmann, Marcin Wawrzyniak, Emilie Gueguen, Maria R. Walker, Yasser Morsy, Kirstin Atrott, Marijn Wilmink, Luise Linzmeier, Marianne R. Spalinger, Sophie Holowacz, Anne Leblanc, Michael Scharl

**Affiliations:** 1 Department of Gastroenterology and Hepatology, University Hospital Zurich, University of Zurich, 8091 Zurich, Switzerland; anna.niechcialtsianas@usz.ch (A.N.); marlene.schwarzfischer@usz.ch (M.S.); paulina.wawrzyniak@usz.ch (P.W.); maditaelena.determann@usz.ch (M.D.); doris.poehlmann@usz.ch (D.P.); marcin.wawrzyniak@usz.ch (M.W.); emilie.gueguen@usz.ch (E.G.); maria.walker@usz.ch (M.R.W.); yasser.morsy@usz.ch (Y.M.); kirstin.atrott@usz.ch (K.A.); marijn.wilmink@usz.ch (M.W.); luise.linzmeier@usz.ch (L.L.); marianne.spalinger@usz.ch (M.R.S.); 2 PiLeJe Laboratoire, 49270 Paris, France; s.holowacz@pileje.com (S.H.); a.leblanc@pileje.com (A.L.)

**Keywords:** colorectal cancer, probiotics, *Lactobacillus*, *Bifidobacterium*, anti-tumor activity

## Abstract

Colorectal cancer (CRC) is a leading cause of cancer-related mortality worldwide with limited treatment options for advanced disease stages. Growing evidence implicates the gut microbiota in CRC pathogenesis, prompting interest in probiotics as a potential therapeutic strategy. In this study, we evaluated the effects of two probiotic compositions, CI (a mix of *lactobacilli* and *bifidobacteria*) and CII (*bifidobacteria alone*), in two murine CRC models: the orthotopic MC-38 cecum injection model and the inflammation-driven azoxymethane/dextran sodium sulfate (AOM/DSS) model. CI showed significant anti-tumor effects in the orthotopic model, reducing tumor weight and volume, which was, however, not associated with robust immune activation, suggesting microbiota-driven mechanisms. In contrast, CII was more effective in the AOM/DSS model, reducing colonic inflammation and completely preventing tumor development. Our study demonstrates that probiotics might have great therapeutic potential via modulation of the gut microbiota, and they can exert anti-tumor effects in murine models of CRC with distinct compositions showing differential efficacy depending on the model. CI stabilized the gut microbiome and inhibited pro-tumorigenic taxa in the MC-38 cecum injection model, while CII exhibited anti-inflammatory properties in the AOM/DSS model, highlighting the potential of probiotics as context-specific interventions for CRC. These findings contribute to the growing body of evidence supporting microbiota-targeted strategies in oncology and their relevance for therapeutic applications.

## 1. Introduction

Colorectal cancer (CRC) is the third most commonly diagnosed cancer worldwide with more than 1.9 million new cases annually [1]. It is also the second leading cause of cancer-related death, accounting for over 900,000 deaths per year [2]. Despite recent advancements in diagnostic and therapeutic strategies, the prognosis for advanced-stage CRC remains poor, underscoring the need for novel preventive and therapeutic approaches [3]. The etiology of CRC is multifactorial, involving genetic, environmental, and lifestyle factors with growing evidence implicating the gut microbiota as a key contributor to its pathogenesis [4]. Emerging evidence suggests that the gut microbiota plays a pivotal role in CRC pathogenesis, influencing carcinogenesis through mechanisms such as inflammation, immune modulation, and the production of carcinogenic metabolites. Consequently, modulating the gut microbiota has been proposed as a potential strategy for CRC prevention and treatment [5,6,7].

Probiotics, defined as live microorganisms that confer health benefits to the host when administered in adequate amounts [8], have gained considerable attention for their potential to modulate the gut microbiota and exert protective effects against various diseases, including CRC [9]. Mechanistically, probiotics may exert anticancer effects by restoring microbial balance, promoting the mucosal barrier, modulating inflammation, and producing metabolites such as short-chain fatty acids that possess anti-inflammatory and antiproliferative properties [10,11,12]. Despite these promising mechanisms, the precise role of probiotics in CRC and their impact on tumor development remain poorly understood and are the subject of ongoing research [13].

Animal models, particularly murine models, have proven invaluable in elucidating the complex interactions between probiotics, the gut microbiota, and colorectal carcinogenesis. These models provide a controlled environment to investigate the molecular and cellular mechanisms underlying probiotic effects, offering valuable insights into their potential translational applications in humans [14,15]. In this study, we aimed to evaluate the effects of probiotic administration on cancer development in two well-established murine models of CRC, namely the orthotopic cecum injection model and the azoxymethane/dextran sodium sulfate (AOM/DSS) inflammation-mediated model that mirrors CRC development observed in colitis-associated cancer. By examining tumor incidence and progression, as well as the intestinal microbiome, we sought to shed light on the therapeutic potential of probiotics as a preventive strategy against CRC and contribute to the growing body of evidence supporting microbiota-based interventions in oncology.

## 2. Results

### 2.1. Administration of Probiotics Affects the Gut Microbiome of Healthy Mice

**Figure 1:**
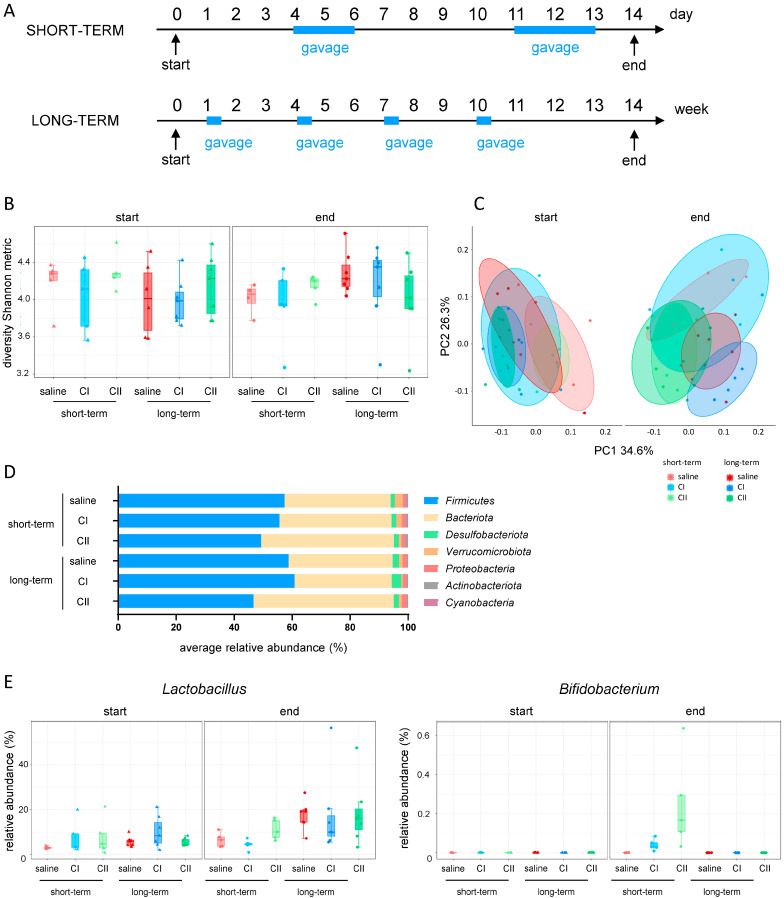
Effects of probiotic administration on intestinal microbiota in healthy mice. (**A**) Experimental design: mice received composition I (CI), composition II (CII), or saline for short-term (2 cycles over 2 weeks) or long-term (4 cycles over 14 weeks), once daily by oral gavage; (**B**) alpha diversity (Shannon metric index); (**C**) beta diversity (weighted UniFrac); (**D**) relative abundance of phyla at the end of experiment; (**E**) probiotic abundance: *Lactobacillus* and *Bifidobacterium* genus.

In an initial step, the impact of the probiotic compositions (mixes) on the microbiota of healthy mice was investigated. For this purpose, composition I (CI) and composition II (CII) were administered in short-term experiments (two cycles of daily gavages for 3 days within 2 weeks) and long-term experiments (four cycles of daily gavages for 3 days within 14 weeks). In both experiments, control groups received saline gavages only (Figure 1A). The alpha diversity Shannon metric score showed no significant differences either at the beginning or the end of the experiment, neither between the treatment groups nor in relation to the duration of administration (Figure 1B). Weighted UniFrac beta diversity revealed close clustering among all groups at the beginning of the experiment. However, the saline-treated control animals started to diverge over time, while the CI and CII-treated animals remained more closely clustered (Figure 1C, Table S1). At the phylum level, minor changes were observed in CI-treated mice, including an increase in the abundance of *Firmicutes* and *Desulfobacteriota* following long-term administration. Moreover, in CII-treated mice, an effect of the microbial dominance shifted from *Firmicutes* toward *Bacteriota*, which was more pronounced after long-term exposure (Figure 1D). Finally, assessing the abundance of the administered probiotics (Figure 1E) revealed that *Lactobacillus* had already colonized the intestine of mice at the beginning of the experiment with its abundance increasing over time in all groups. Whilst *Bifidobacterium* was completely absent at the beginning of the administration experiments, it evidently increased only following short-term administration of CI and CII. This may be attributed to the 4-week period without treatment after the last gavage in the long-term administration experiment, suggesting that the successful maintenance of intestinal probiotic bacteria requires frequent and continued administration.

### 2.2. Composition I Has Greater Anti-Tumor Potential than Composition II in the Orthotopic CRC Model

**Figure 2:**
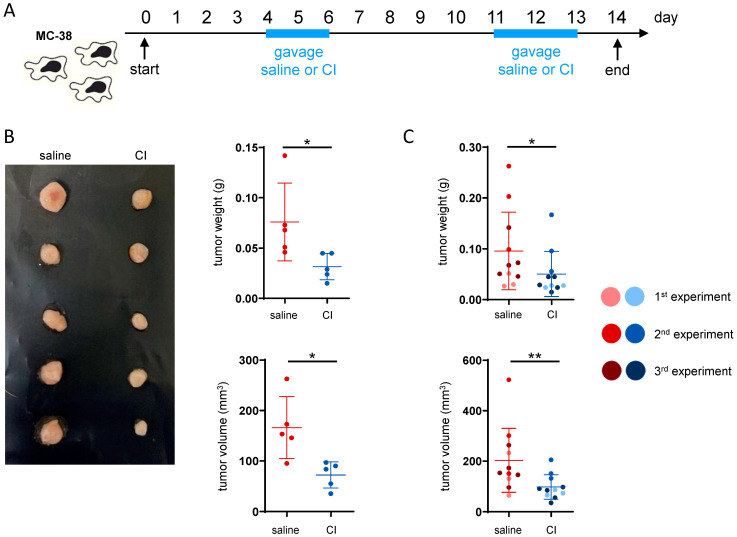
Composition I demonstrates higher anti-tumor potential than composition II in an orthotopic CRC model. (**A**) Experimental design: CRC was induced by implanting the MC-38 cells into the cecum wall. Composition I (CI) was administered in two cycles (days 4–6 and 11–13) once daily by oral gavage, while control mice received saline; (**B**) representative picture and raw tumor data from a single experiment; (**C**) tumor weight and volume—pooled data from three independent experiments; * *p* < 0.05, ** *p* < 0.01.

Given the observed impact on the intestinal microbiome composition after the administration of our two probiotics compositions, we evaluated their effect on CRC development. For this purpose, the orthotopic MC-38 CRC cecum injection model was used. In this model, MC-38 colon adenocarcinoma cells are injected into the cecum wall of C57BL/6 mice. CI and CII were administered in two cycles of daily gavages—first from day 4 to 6 and secondly from day 11 to 13 (Supplementary Figure S1A). Control animals received gavages of saline only. In two initial independent experiments, both CI and CII impacted tumor development, as evidenced by decreases in tumor weight and volume. However, CI seemed to induce a more pronounced effect (Supplementary Figure S1B,C). Therefore, we further investigated the anti-tumor potential of CI in the cecum injection model in three independent follow-up experiments, using the same administration scheme and experimental design (Figure 2A). Again, a clear reduction in tumor weight and volume (Figure 2B) was observed, which became statistically significant when the data from all three consecutive experiments were pooled (Figure 2C).

### 2.3. The Anti-Tumor Activity of CI Does Not Appear to Be Immune-Mediated

**Figure 3:**
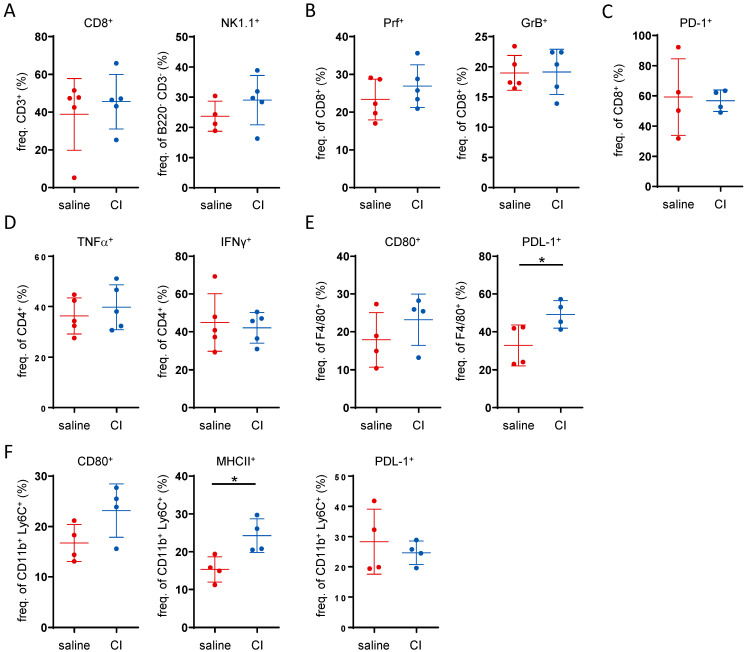
The anti-tumor activity of Composition I is not primarily driven by a clear immune-mediated mechanism. Flow cytometry data showing the frequency on intra-tumoral immune cells: (**A**) CD8^+^ cytotoxic T-cells, and NK1.1^+^ NK-cells, (**B**) perforin and granzyme B producing CD8^+^ T-cells, (**C**) PD-1 expressing CD8^+^ T-cells, (**D**) TNF-α and IFN-γ producing CD4^+^ T-cells, (**E**) CD80 and PDL-1 expressing F4/80 macrophages and (**F**) CD80, MHCII and PDL-1 expression in CD11b^+^Ly6C^+^ monocytes. * *p* < 0.05.

Given that CI demonstrated a clear anti-tumor potential, we aimed to assess whether the observed effect was immune-mediated. To this end, the proportions and activation status of intra-tumoral immune cells was analyzed by flow cytometry (gating strategies are shown in the Supplementary Figure S2). With regard to the overall frequency of B-cells, T-cells and NK cells, only a minor increase in CD8^+^ cells and NK1.1^+^ cells in the tumor tissue was visible in the CI-treated group (Figure 3A and Figure S3A). Among the CD8^+^ cytotoxic T-cells, there was a slight trend toward an increase in perforin-positive (Prf^+^) cells. However, no differences were observed in granzyme B (GrB^+^) levels (Figure 3B) or T-cell exhaustion, as indicated by PD-1 staining (Figure 3C). In pro-inflammatory CD4^+^ T-cells, a slight tendency toward an increase in TNF-α but not IFN-γ (Figure 3D) levels was observed. In CD8^+^ T-cells, no difference in TNF-α or IFN-γ levels was observed upon CI administration. In fact, nearly all CD8^+^ T-cells were positive for both cytokines regardless of CI administration (Supplementary Figure S3B). No differences in myeloid cell infiltration into the tumor tissue were observed, as the frequency of neutrophils, macrophages, monocytes and dendritic cells did not differ between the treatment groups (Supplementary Figure S3C). However, in CI-treated animals, there was an increase in CD80-expressing macrophages and a significant induction of PDL-1 production (Figure 3E). In monocytes, increased surface expression levels of CD80 and MHCII were observed in CI-treated animals, while PDL-1 expression was slightly reduced (Figure 3F). To further explore potential mechanisms, untargeted metabolomics analysis of the serum was conducted alongside flow cytometry analysis of the immune cells. The relatively close clustering of both treatment groups in the PCA plot suggests that the overall differences in their serum metabolomic profiles were minimal (Supplementary Figure S4, Table S2). The butyrate level was not altered or correlated with tumor load (Supplementary Figure S4). Collectively, these findings suggest that the anti-tumor activity of CI is not primarily driven by immune-mediated or metabolomic alterations.

### 2.4. Administration of Composition I Modulates the Gut Microbiome to Promote a Healthy Microbial Environment in the MC-38 Tumor Injection Model

**Figure 4:**
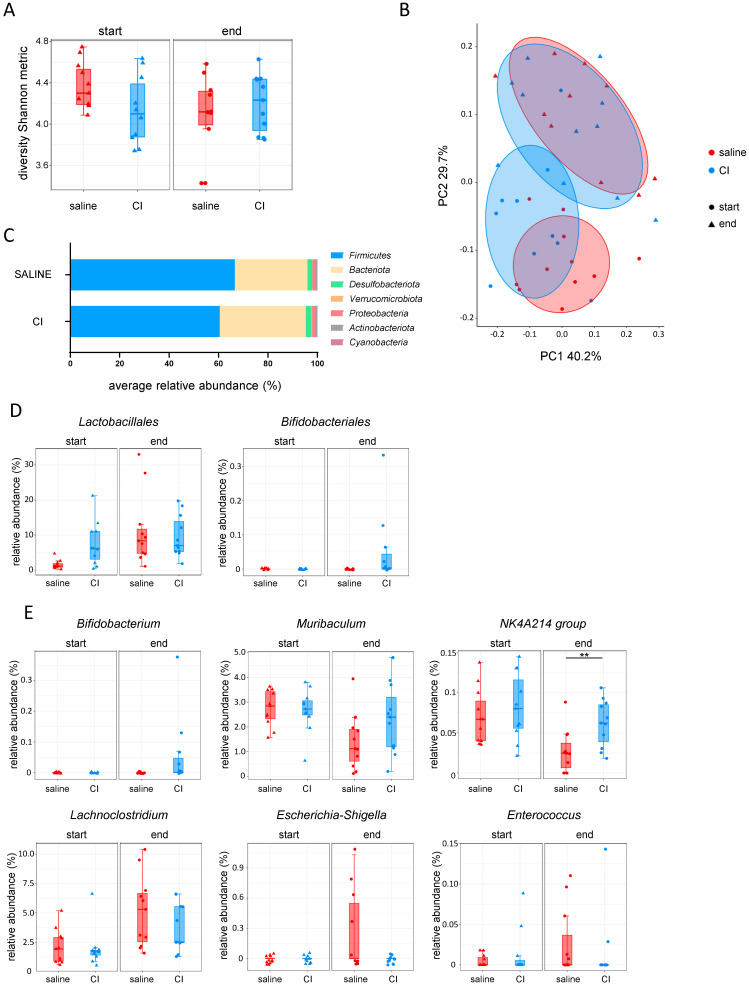
Composition I administration shapes the gut microbiome to foster a healthy microbial environment in the MC-38 tumor model. (**A**) Alpha diversity (Shannon metric index); (**B**) beta diversity (weighted UniFrac); (**C**) relative abundance of phyla at the end of experiment; (**D**) probiotic abundance: *Lactobacillus* and *Bifidobacterium*; (**E**) genus-level abundance of *Bifidobacterium*, *Muribaculum*, *NK4A214 group*, *Lachnoclostridium*, *Escherichia-Shigella*, and *Enterococcus*.

To study the impact of CI on the intestinal microbiome in the context of CRC, 16S sequencing of fecal samples (sampled at the start and at the end of each experiment) from control and CI-treated mice was performed, combining data from the three cecal injection experiments with CI. At the start of the experiment, the CI group exhibited slightly lower Shannon diversity indices. However, by the end, alpha diversity increased in CI-treated mice, while it decreased in the saline group (Figure 4A). In terms of beta diversity, there was a substantial overlap between the saline and CI groups at the start of the experiment. However, by the end, the saline group became more distinct, indicating that probiotic administration altered the microbial composition over time (Figure 4B, Table S1). At the phylum level, *Firmicutes* were less abundant in the CI-treated group, while *Bacteroidota* increased, indicating a shift in microbiome composition (Figure 4C). When examining the abundance of the administered probiotics, *Lactobacillales* abundance increased over time in both groups, whereas *Bifidobacteriales* appeared only in the CI-treated animals (Figure 4D). In CI-treated mice, *Bifidobacterium* was clearly present at the genus level, along with the stabilization of *Muribaculum* and the *NK4A214 group* (Figure 4E). The abundance of these genera, which are highly associated with a healthy gut microbiome, notably decreased in the saline-treated group during CRC progression. Furthermore, CI administration inhibited the increase in *Lachnoclostridium*, was associated with pro-tumorigenic processes in CRC, and prevented the rise of pathogenic bacteria such as *Escherichia-Shigella* and *Enterococcus* (Figure 4E).

### 2.5. Composition II Exhibits Greater Anti-Tumor Potential in the AOM/DSS Inflammation-Mediated CRC Model

**Figure 5:**
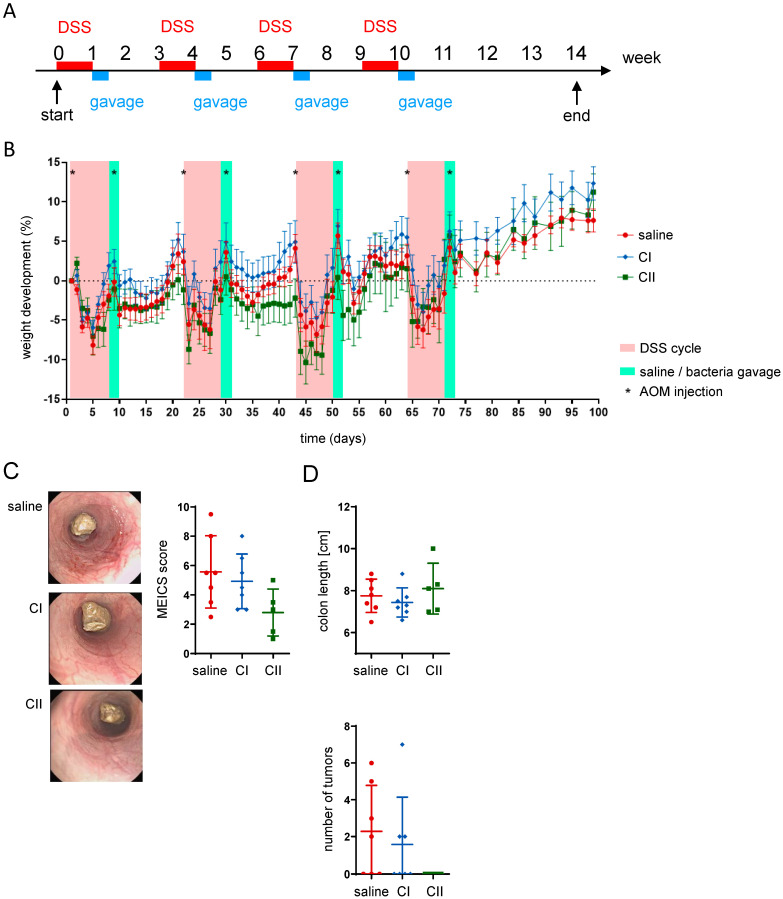
Composition II demonstrates higher anti-tumor potential in the AOM/DSS inflammation-mediated CRC model. (**A**) Experimental design: colon tumors were induced by co-administration of DSS in drinking water (four 1-week cycles followed by 2 weeks of only water recovery period) and AOM intraperitoneal injections on days 1 and 9 of each cycle; mice received daily gavages of saline, composition I (CI), or composition II (CII) for 3 days at the end of each DSS cycle; (**B**) weight development of mice throughout the experiment; (**C**) representative pictures from mouse colonoscopy performed at the end of the experiment and the respective murine endoscopic index of colitis severity (MEICS) score; (**D**) colon length and number of tumors within the colon.

To validate the anti-tumor potential of the probiotics, the therapeutic effects of both compounds were tested in the azoxymethane/dextran sodium sulfate (AOM/DSS) inflammation-dependent model of colitis-associated cancer. In this model, tumor development is induced by administering the chemical carcinogen AOM in combination with DSS, which triggers chronic intestinal inflammation. The experiment involved four cycles of DSS administration in drinking water, each lasting 7 days, which was followed by a 14-day recovery period with regular drinking water (no DSS). After the final DSS cycle, the animals underwent a 4-week recovery period. In each cycle, AOM was administered via intraperitoneal injection on days 1 and 9 (Figure 5A). At the end of each DSS cycle, mice received daily gavage of saline, CI or CII for 3 consecutive days. Throughout the experiment, mice in each group showed weight loss in response to DSS and AOM treatments but quickly regained weight during phases with regular drinking water (Figure 5B). To evaluate the impact of probiotics on intestinal inflammation, colonoscopy was performed at the end of the experiment. The murine endoscopic index of colitis severity (MEICS) score indicated that CI had a limited effect, while CII reduced colonic inflammation (Figure 5C). While both compositions had only little effect on colon length (which typically shortens upon the presence of intestinal inflammation), mice treated with CII did not develop tumors in the colon (Figure 5D).

**Figure 6:**
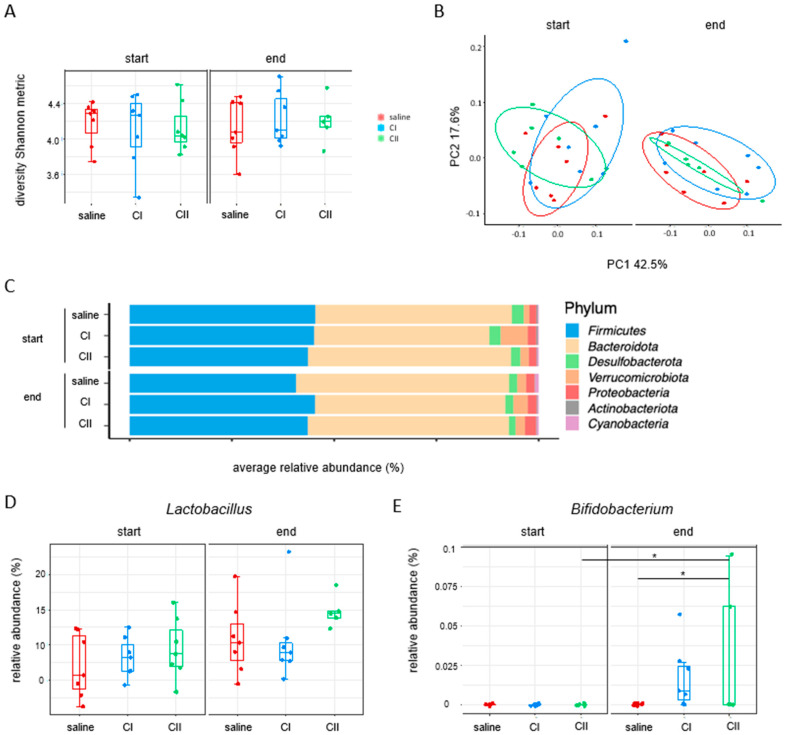
Impact of composition I and composition II on the fecal microbiome in the AOM/DSS inflammation-mediated CRC model. (**A**) Alpha diversity (Shannon metric index); (**B**) beta diversity (weighted UniFrac); (**C**) relative abundance of phyla at the start and at the end of the experiment; (**D**,**E**) relative abundance of probiotics: *Lactobacillus* and *Bifidobacterium*. * *p* < 0.05.

When examining the fecal microbiome composition after probiotics treatment by 16s rRNA sequencing, no significant differences were observed in the abundance of *Lactobacillus*. In contrast, *Bifidobacterium* increased with the administration of both compositions (Figure 6).

## 3. Discussion

Our study highlights the potential of probiotics in the prevention of CRC through the modulation of the gut microbiota while also revealing key differences in their effectiveness depending on microbial composition and disease model. The probiotic composition I (CI, *lactobacilli* and *bifidobacteria* mix) and II (CII, *bifidobacteria*) demonstrated distinct effects in altering the microbial composition and influencing tumor progression in the two well-established murine CRC models that were used here. The alterations observed in the gut microbiota following probiotic administration indicate a potential to promote a potentially healthier microbial environment.

The administration of both CI and CII resulted in distinct microbial shifts. CI demonstrated the ability to increase the abundance of *Bifidobacterium* and other health-associated genera such as *Muribaculum* and *Lachnospiraceae NK4A214 group* while reducing pathogenic taxa including *Escherichia-Shigella* and *Enterococcus*. Together with the tumor-reducing effects that we observed upon the administration of CI, this aligns with the hypothesis that a balanced microbiota might mitigate CRC-associated dysbiosis [16,17]. Furthermore, the enhanced abundance of beneficial taxa in CI-treated mice, combined with the inhibition of pro-tumorigenic genera such as *Lachnoclostridium*, further supports the role of probiotics in reshaping the gut microbiome to counteract carcinogenesis [16]. These findings are particularly relevant given the pressing need for novel therapeutic intervention strategies and the growing interest in microbiota-based interventions for CRC prevention [18].

Notably, CI treatment was associated with an increased abundance of *Lachnospiraceae NK4A214*, which is a known butyrate-producing group. While our untargeted metabolomics did not show significant differences in systemic metabolite profiles, we acknowledge that targeted short-chain fatty acid (SCFA) quantification, particularly of butyrate in fecal or cecal samples, could provide more specific insight. This remains an important avenue for future investigation, as local SCFA production may contribute to the tumor-suppressive effects observed with CI.

In addition, our data demonstrate a clear distinction in the anti-tumor potential of the two compositions depending on the CRC model employed. In the orthotopic tumor injection model, CI exhibited significant anti-tumor activity, which was evidenced by reduced tumor weight and volume. In contrast, CII demonstrated superior efficacy in the AOM/DSS inflammation-mediated CRC model, effectively reducing colonic inflammation and preventing tumor development. The differential efficacy of CI and CII across CRC models highlights the importance of tailoring probiotic interventions to specific disease contexts. The ability of CI to promote a healthy microbial environment and reducing pathogenic bacteria growth is particularly relevant in contexts where microbiota dysbiosis is associated with tumor progression. Conversely, the anti-inflammatory properties of CII render it a promising candidate for addressing inflammation-driven CRC. Although we did not perform in-depth immune or metabolite profiling in the CII group, the rapid clinical improvement suggests early anti-inflammatory effects. Whether these effects are mediated by microbial changes or through host responses to probiotic-derived components remains to be clarified. We recognize that the lack of mechanistic data for CII is a limitation and have now outlined this as a priority for future research. Overall, these findings emphasize the importance of further investigation into the mechanistic underpinnings of probiotic action, particularly the role of microbial metabolites and their interaction with host pathways.

Our results suggest that the observed anti-tumor effects of CI in the MC-38 injection model may be mediated through microbiota-driven pathways rather than direct immune or metabolomic modulation. Despite slight trends in the induction of cytotoxic CD8^+^ T-cells and macrophage activation, no significant changes in cytokine production, cytotoxicity or immune activation were observed. Furthermore, serum metabolomics analysis revealed no significant differences between the CI-treated and control groups with close clustering of the two groups in PCA plots, indicating subtle or minimal differences in the metabolomic profiles.

While our study provides evidence of the anti-tumor potential of probiotics, there are also some limitations. One notable limitation was the relatively small group size in the orthotopic MC-38 cecum injection model which was accounted to logistical challenges related to surgical cell implantation procedures, such as the availability of trained personnel and the need to ensure consistent, precise operations, which restricted the number of animals that could be treated simultaneously. While the results observed in this model are promising and consistent across experiments, future studies should aim to include larger cohorts to strengthen statistical power and enhance the robustness of the findings. Secondly, the reduced tumor size observed in treated animals, while indicative of therapeutic efficacy, resulted in less material available for subsequent analyses, which limited the extent of downstream investigations, such as immune cell characterization and more comprehensive molecular profiling. Additionally, variability in immune responses between experiments was observed, highlighting the complexity of probiotic–host interactions and the need for more in-depth investigations into their molecular mechanisms. The long-term effects of probiotics on CRC progression and recurrence remain to be explored. Lastly, translating these findings to human CRC requires clinical validation, as murine models may not fully recapitulate the complexities of human disease.

Importantly, the anti-tumor effects of both compositions were observed alongside distinct microbial patterns, supporting the hypothesis that probiotics may function through microbiota-driven mechanisms, which are shaped by both composition and disease context. However, our microbiota analyses were limited to initial and final time points. While this allowed us to assess global shifts, it likely missed dynamic or transient microbial changes during disease progression, particularly in the long-term AOM/DSS model. We now acknowledge this as a limitation and emphasize the need for intermediate time-point sampling in future longitudinal studies to better capture microbiota–host interactions over time.

Despite the limitations mentioned, this study provides several novel contributions. First, to our knowledge, this is among the first direct comparisons of two compositionally distinct probiotic blends across two CRC models, revealing model-specific efficacy. Second, we challenge the common assumption that probiotics exert anti-tumor effects primarily through immune activation. In the case of CI, our data suggest a microbiota-centric mode of action, which may operate independently of systemic immune responses. Finally, the differential response to CI and CII across models underscores the need for more personalized and context-aware strategies in microbiota-based therapy.

Future research should investigate the combinatorial effects of probiotics with other therapeutic modalities, such as chemotherapy and immunotherapy, since the integration of probiotics into existing treatment regimens may enhance therapeutic efficacy while minimizing side effects.

In conclusion, our study reinforces the therapeutic potential of probiotics in CRC prevention and treatment, highlighting the importance of model-specific strategies. It contributes to the growing evidence supporting microbiota-targeted interventions in oncology, encourages further research into the mechanistic pathways involved, including microbial metabolites and host interactions, and lays the groundwork for integrating probiotics into personalized medicine frameworks. More personalized approaches to probiotic therapy, guided by individual microbiome profiles, hold promise for optimizing outcomes in the prevention and treatment of CRC in the future.

## 4. Materials and Methods

### 4.1. Animal Experiments

All animal experiments were performed according to the Swiss Animal Welfare Legislation and approved by the local veterinary office, the Veterinary Office of the Canton Zurich (Licenses ZH153-2020 and ZH154-2020). Mice: In all studies, 12-week-old C57BL/6J female wild-type (WT) mice purchased from Janvier Labs, Le Genest-Saint-Isle, France, were used for the experiment. Upon arrival at our facility, the mice were given 2 weeks of acclimatization time. The mice were kept in a specific pathogen-free (SPF) facility with chow and water ad libitum. Study design: The primary outcome of the study was either the tumor volume measured in mm^3^ or number of colon tumors (AOM/DSS model). Randomization: Prior to the experiment, the mice were randomized using a random number generator. Blinding: Treatment administration was conducted blindly by using allocation groups to ensure that all animals in the experiment were handled, monitored, and treated the same way. Sample collection at the end of the experiment and sample analysis were conducted in a blinded manner. Statistics: Analysis was performed using the GraphPad Prism 10 software. Sample size calculations: The tumor cells were injected, and the bacteria were administered via oral gavage for each mouse individually; thus, each mouse was considered an experimental unit. Student’s *t*-test was performed when comparing only two conditions, while a one-way analysis of variance (ANOVA) test was chosen when comparing more than 3 conditions. Data are presented as means ± standard deviation. *p*-values lower than 0.05 were considered statistically significant.

### 4.2. Orthotopic Cecum Injection Model

MC-38 cells (isolated and provided from Prof. Dr. Lubor Borsig, University Zürich, Zürich, Switzerland): 300,000 cells in high-glucose DMEM (Gibco, Waltham, MA, USA) medium mixed 1:1 with Matrigel (Corning, Corning, NY, USA) were injected into the cecum wall during a surgical procedure under isoflurane anesthesia. Prior to the surgery, mice received a subcutaneous injection of the painkiller Finadyne (MSD Animal Health, Rahway, NJ, USA). After the surgery, an analgesia mix consisting of tramadol (Tramal, Grünenthal, Aachen, Germany) and paracetamol (Dafalgan, UPSA, Rueil Malmaison, France) was administered in the drinking water for 2–3 days. Mice were sacrificed 14 days post tumor cells injection.

### 4.3. AOM/DSS Model

Four cycles of dextran sodium sulfate (DSS, Fisher Scientific/MP Biomedicals, Irvine, CA, USA) were applied; in each, 1–1.5% DSS was administered in the drinking water for 7 days, which was followed by 14 days of recovery with drinking water only. Azoxymethane (AOM, Sigma-Aldrich/Merck, St. Louis, MO, USA) was injected intraperitoneally (1 mg/kg in 100 µL PBS) on the 1st and 9th day of each DSS cycle. After the final DSS cycle, mice received normal drinking water for 4 weeks before sacrifice.

### 4.4. Probiotics

Composition I (*Bifidobacterium lactis PI61*, *Bifidobacterium bifidum PI62*, *Bifidobacterium lactis PI63*, *Bifidobacterium breve PI64*, *Lactobacillus acidophilus PI1*, *Lactobacillus rhamnosus PI28*, *Lactobacillus gasseri PI17*, and *Lactobacillus acidophilus PI4* mix, abbreviated as CI) and composition II (*Bifidobacterium lactis PI61*, *Bifidobacterium bifidum PI62*, *Bifidobacterium lactis PI63*, and *Bifidobacterium breve PI64 mix*, abbreviated as CII) were provided by PiLeJe (Paris, France) and administered by oral gavage (10 × 10^8^ CFU per dose). For every administration, probiotics were freshly prepared by reconstituting frozen lyophilizates in saline. In the short-term administration experiment and in cecum injection experiments, bacteria were applied once daily in two periods: days 4–6 and days 11–13. In the long-term administration experiment and in the AOM/DSS experiment, bacteria were applied once daily for the first 3 days of weeks 1, 4, 7 and 10 of the experiment. In the control groups, mice received gavages of saline instead of bacteria.

### 4.5. Clinical Readouts

In every cecum injection experiment, the obtained tumors were weighted and measured. The tumor volume was calculated according to the following formula: $V=\frac{4}{3}\times 3.14\times \frac{a}{2}\times \frac{b}{2}\times \frac{c}{2}$, where “V” is the tumor volume, and “a”, “b”, “c” are the tumor dimensions. In the AOM/DSS experiment colon length was measured, number of tumors within the colon was counted and colonoscopy was performed using a mouse endoscope (Karl Storz, Tuttlingen, Germany) and evaluated using the murine endoscopic index of colitis severity (MEICS score) as described by Becker et al. [19], which consists of scoring for the thickening and granularity of the mucosal wall as well as alterations in vascularity, stool consistency and the presence of fibrin.

### 4.6. Tumor Processing for Flow Cytometry

Upon removal, tumors were placed in PBS and kept on ice prior to further processing. Once all the tumors were collected, each tumor was cut into pieces using small size surgical scissors. Tumors were then enzymatically digested using the Tumor Dissociation Kit Mouse (Miltenyi Biotec, Bergisch Gladbach, Germany) according to the manufacturer’s instructions.

### 4.7. Flow Cytometry

Single-cell suspensions obtained from each tumor were divided into two different sets for flow cytometry staining. One set was re-stimulated using a cocktail for immune cell activation and subsequently stained for cytokine’s evaluation, while the other set was stained immediately after tissue processing. Re-stimulation was performed using 50 ng/mL PMA (Sigma Aldrich, St. Louis, MO, USA), 1 µg/mL ionomycin (Sigma Aldrich) and 1 µg/mL brefeldin A (Sigma Aldrich) in RPMI 1640 medium (Thermo Fisher Scientific, Waltham, MA, USA). Cells were re-stimulated in 12-well cell culture plates (TPP) for 3 h in 5% CO_2_. First, viability staining was performed using Zombie NIR (BioLegend) for 30 min at room temperature in the dark. Cells where then washed with PBS and stained with TruStain FcS™ PLUS (BioLegend) for 5 min at 4 °C in the dark. Further, cells were washed with PBS and stained with anti-CD4-BUV563 (BD Biosciences, Franklin Lakes, NJ, USA), anti-NK1.1-BUV395 (BD Biosciences, Franklin Lakes, NJ, USA), anti-CD3-BV785 (BioLegend, San Diego, CA, USA), anti-CD45-PB (BioLegend, San Diego, CA, USA), anti-CD45R(B220)-PE-Cy5 (Thermo Fisher Scientific, Waltham, MA, USA), and anti-CD8-PE-CF594 (BD Biosciences, Franklin Lakes, NJ, USA) antibodies for 15 min at 4 °C in the dark. After washing with MACS buffer (autoMACS™ Running Buffer, Miltenyi Biotec, Bergisch Gladbach, Germany), cells were permeabilized for 20 min at 4 °C in the dark using the BD Cytofix/Cytoperm (BD Biosciences, Franklin Lakes, NJ, USA) kit and stained intracellularly using anti-CD4-BUV563 (BD Biosciences, Franklin Lakes, NJ, USA), anti-CD3-BV785 (BioLegend), anti-TNFα-BV650 (BioLegend), anti-IL-17-BV510 (BioLegend), anti-Granzyme-B-PerCP-Cy5.5 (BioLegend), anti-Perforin-FITC (e-Bioscience), anti-IFN-γ-PE-Cy7 (BioLegend), and anti-CD8A-PE-CF594 (BD Biosciences, Franklin Lakes, NJ, USA) antibodies for 30 min at 4 °C in the dark. Finally, cells were washed with BD Perm Wash (BD Biosciences) and kept at 4 °C in 0.1% paraformaldehyde (PFA, Sigma-Aldrich, St. Louis, MO, USA) until acquisition. For the second set of cells without the re-stimulation, cells were processed in the same way; however, the surface staining consisted of the following antibodies: anti-CD80-BUV737 (BD Biosciences, Franklin Lakes, NJ, USA), anti-Ly6G-BUV563 (BD Biosciences, Franklin Lakes, NJ, USA), anti-CD4-BUV496 (BD Biosciences, Franklin Lakes, NJ, USA), anti-CD3-BV785 (BioLegend, San Diego, CA, USA), anti-CD45-BV650 (BioLegend, San Diego, CA, USA), anti-CD11b-BV605 (BioLegend, San Diego, CA, USA), anti-Ly6C-BV570 (BioLegend, San Diego, CA, USA), anti-CD274(PD-L1)-BV480 (BD Biosciences, Franklin Lakes, NJ, USA), anti-F4/80-FITC (BioLegend, San Diego, CA, USA), anti-CD11c-PE-Cy7 (BioLegend, San Diego, CA, USA), anti-CD45R(B220) (Thermo Fisher Scientific, Waltham, MA, USA), anti-CD8A-PE-CD594 (BD Biosciences, Franklin Lakes, NJ, USA), anti-I-A/I-E(MHCII)-AF700 (BioLegend, San Diego, CA, USA) and anti-CD279(PD-1)-APC (BioLegend, San Diego, CA, USA), and anti-CD274(PD-L1)-BV480 (BD Biosciences, Franklin Lakes, NJ, USA). Cells were acquired using the FACSymphony (BD Biosciences, Franklin Lakes, NJ, USA) and the analysis was performed using the FlowJo software (Version 10, FlowJo LLC/BD, Ashland, OR, USA).

### 4.8. Microbiota 16S Sequencing

Fecal samples were collected at the beginning and at the end of each experiment and subsequently stored at −80 °C prior to analysis. DNA extraction and sequencing of the V4 region of 16S ribosomal DNA was performed by Microsynth AG (Balgach, Switzerland). The QIIME2 pipeline was utilized for analyzing 16S rRNA [20]. After assessing data quality, denoising with DADA2 was conducted to merge the paired reads, producing amplicon sequence variants (ASVs) [21]. The alpha rarefaction module was employed to ensure sufficient depth for capturing most features. We then calculated both alpha and beta diversity using the core-metrics-phylogenetic module. For the alpha analysis, we utilized the faith phylogenetic matrix to compute richness and incorporate phylogenetic relationships. For beta diversity, we employed the weighted UniFrac distance matrix to quantify the dissimilarity between communities. Principal coordinate analysis (PCoA) was conducted to enhance the visualization of beta diversity. Taxonomy was assigned to ASVs using the q2-feature-classifier classify-sklearn naïve Bayes taxonomy classifier against the pre-trained Naïve Bayes silva-132-99-nb-classifier, which was trained on Silva (release 132) full-length sequences [22]. The Taxa barplot module was used to visualize the different taxonomy compositions.

### 4.9. Serum Metabolomics

On the last day of the experiment, blood was collected into BD Microtainer tubes with a serum separator and centrifuged at the maximum speed for 5 min at room temperature. The obtained serum was transferred into a 0.5 mL Eppendorf tube and immediately snap frozen in liquid nitrogen, and all samples were stored at −80 °C prior to analysis. Metabolomics analysis was carried out with a high-resolution mass spectrometer (Agilent QTOF 6550, Agilent, Santa Clara, CA, USA), and analysis was performed by MetaboAnalystR package version 4.2 for the statistical analysis [23]. The normalized intensities by the median for each sample were mean-centered and divided by the standard deviation of each variable for all annotated features. A feteroscedastic (two-tail, unequal variance) *t*-test was used to determine significant differences between the groups. In addition, *p*-values were adjusted according to Benjamini–Hochberg, and q-values were adjusted according to Storey and Tibshirani. Principle component analysis (PCA) was used to visualize sample variance. Heatmap visually presented hierarchical clustering using the Euclidean method for distance calculation and Ward’s linkage for clustering.

### 4.10. Statistical Analysis

Analysis was performed using the GraphPad Prism 10 software. Student’s *t*-test was performed when comparing only two conditions, while an ANOVA test with Tukey’s HSD was chosen when comparing more than 3 conditions. Data are presented as means ± standard deviation. *p*-values lower than 0.05 were considered statistically significant.

## Data Availability

The original contributions presented in this study are included in the article. Further inquiries can be directed to the corresponding author.

## Supplementary Materials

The following supporting information can be downloaded at: https://www.mdpi.com/article/10.3390/ijms26094404/s1.
