# Supplementary material for: Probiotic Administration Modulates Gut Microbiota and Suppresses Tumor Growth in Murine Models of Colorectal Cancer
Source: Int J Mol Sci. 2025 May 6;26(9):4404. doi: 10.3390/ijms26094404 (PMC12072948; doi:10.3390/ijms26094404)
Supplement: Supplementary file 1 [file ijms-26-04404-s001.zip › PLJ supp figures_060525.pptx]

## Slide 1
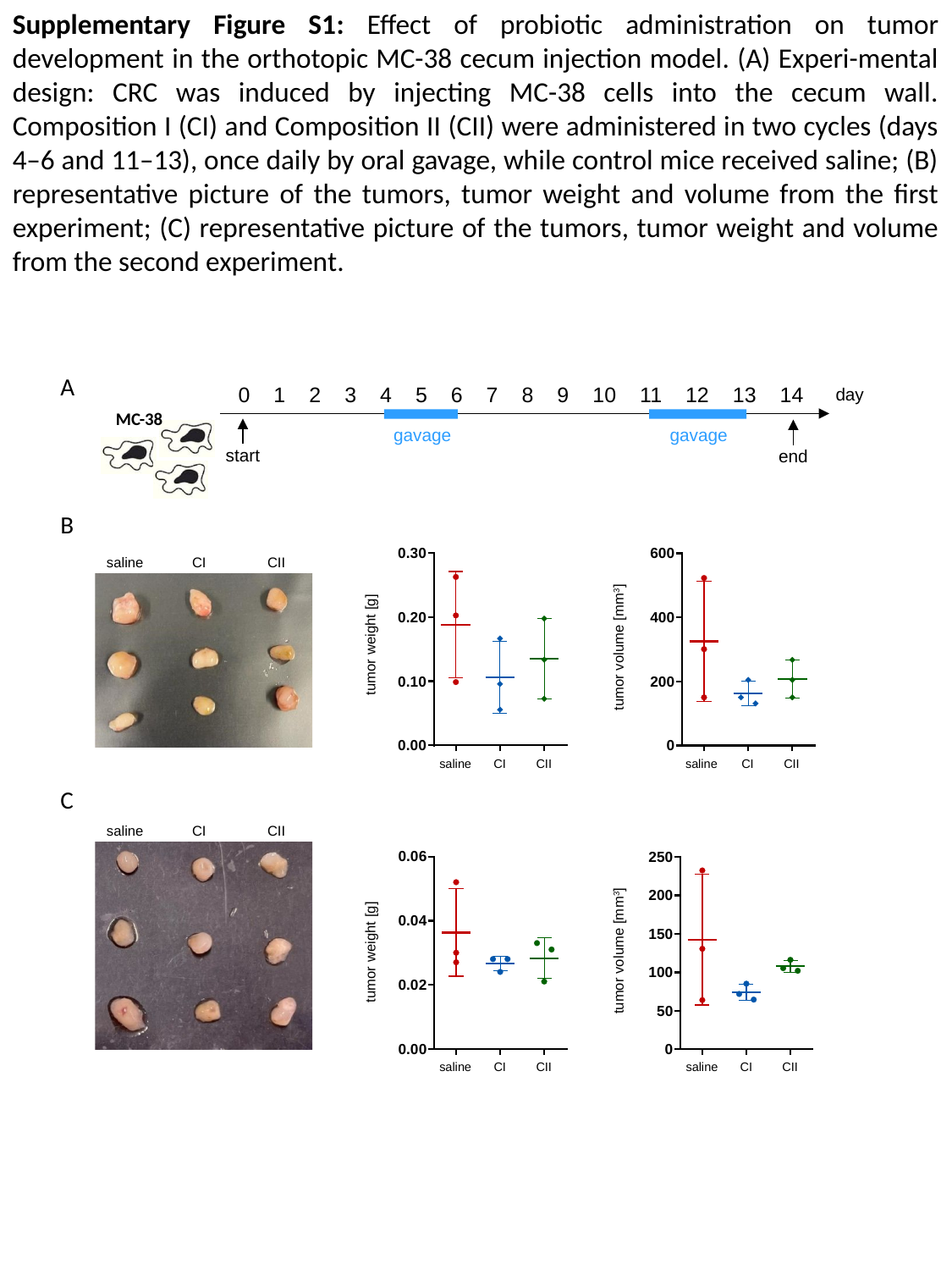

Supplementary Figure S1: Effect of probiotic administration on tumor development in the orthotopic MC-38 cecum injection model. (A) Experi-mental design: CRC was induced by injecting MC-38 cells into the cecum wall. Composition I (CI) and Composition II (CII) were administered in two cycles (days 4–6 and 11–13), once daily by oral gavage, while control mice received saline; (B) representative picture of the tumors, tumor weight and volume from the first experiment; (C) representative picture of the tumors, tumor weight and volume from the second experiment.
A
0 1 2 3 4 5 6 7 8 9 10 11 12 13 14
day
MC-38
gavage
gavage
start
end
B
saline
CI
CII
tumor weight [g]
tumor volume [mm3]
saline
CI
CII
CI
CII
saline
C
saline
CI
CII
tumor volume [mm3]
tumor weight [g]
saline
CI
CII
saline
CI
CII

## Slide 2
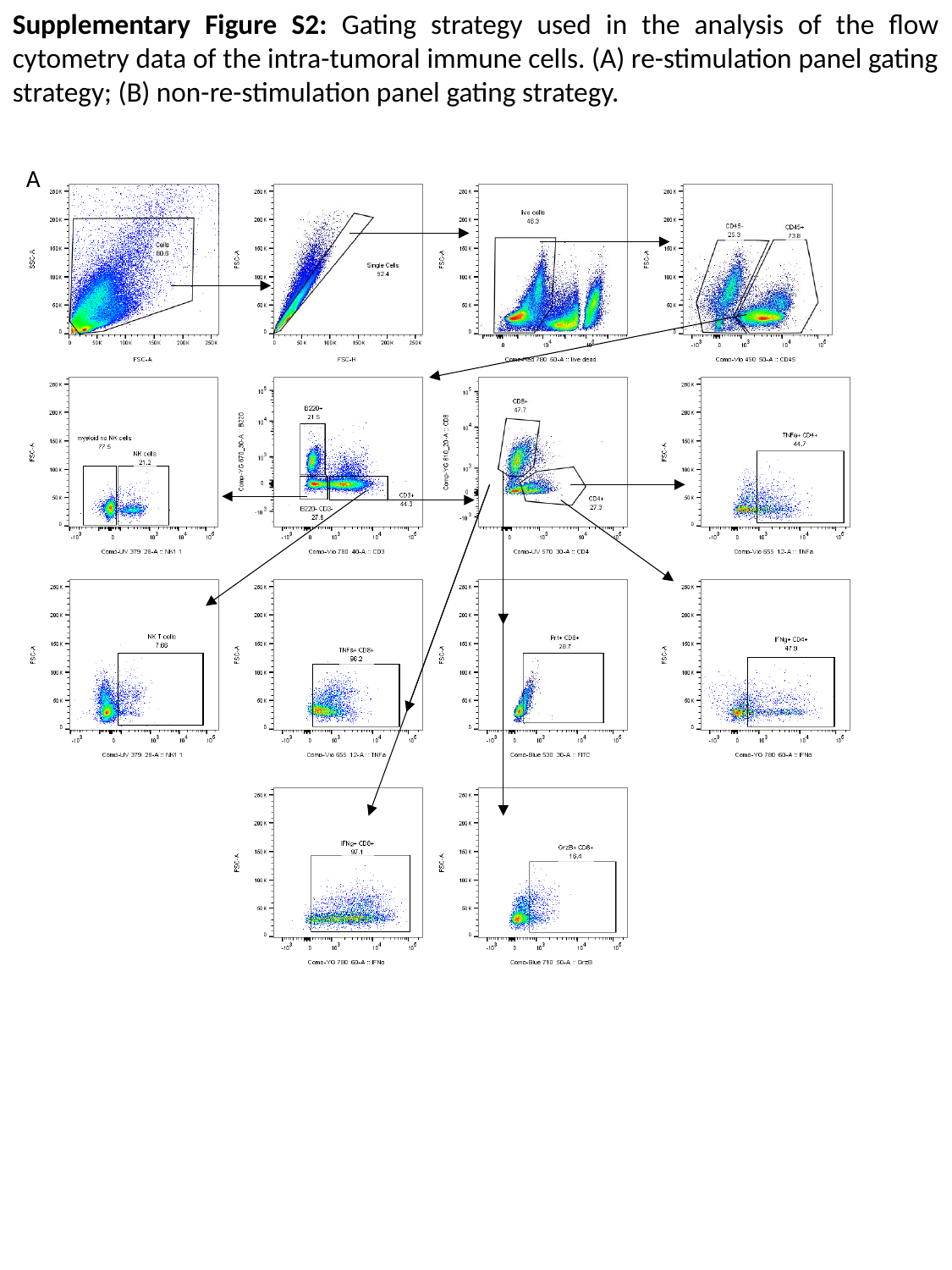

Supplementary Figure S2: Gating strategy used in the analysis of the flow cytometry data of the intra-tumoral immune cells. (A) re-stimulation panel gating strategy; (B) non-re-stimulation panel gating strategy.
A

## Slide 3
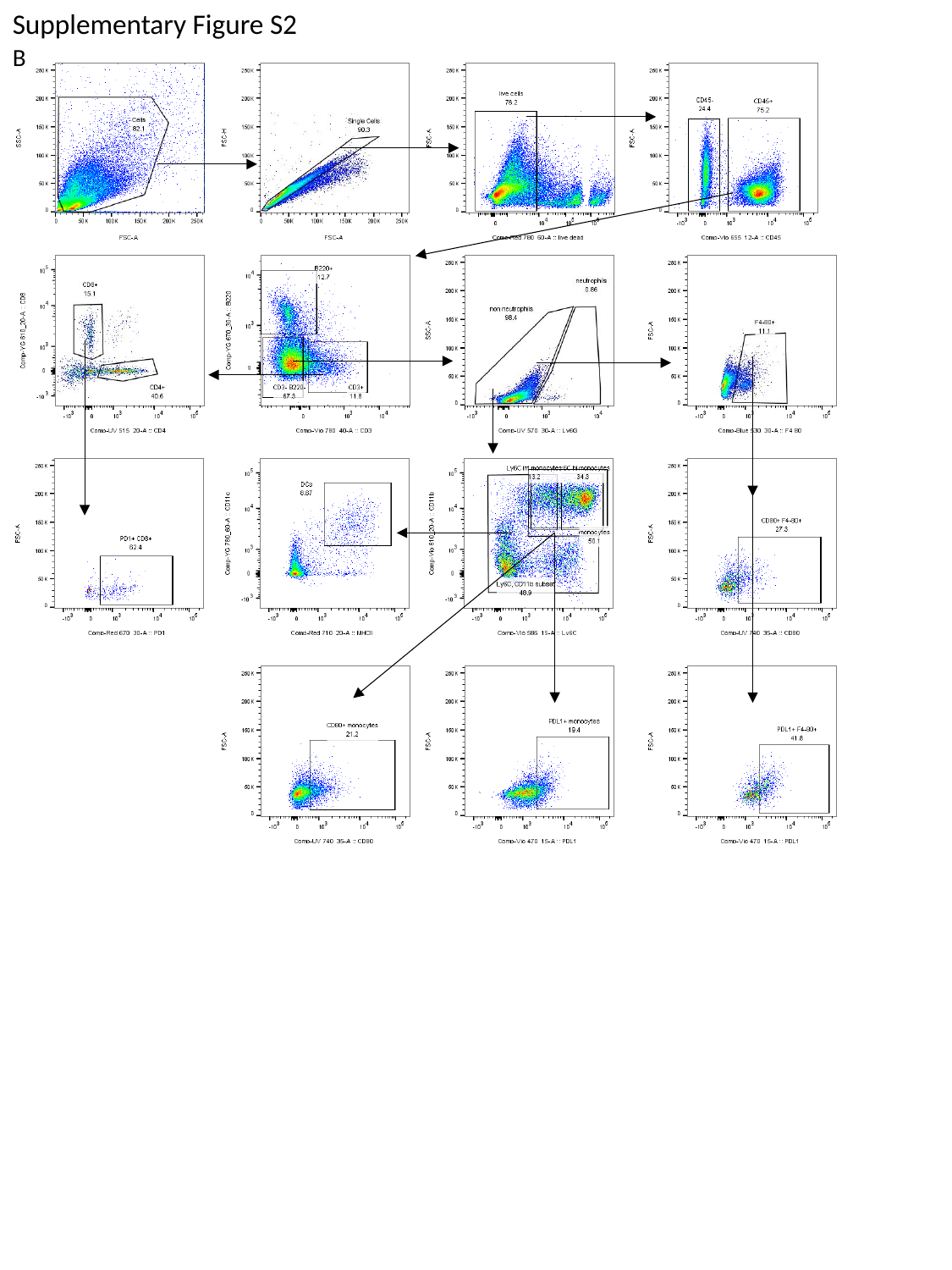

Supplementary Figure S2
B

## Slide 4
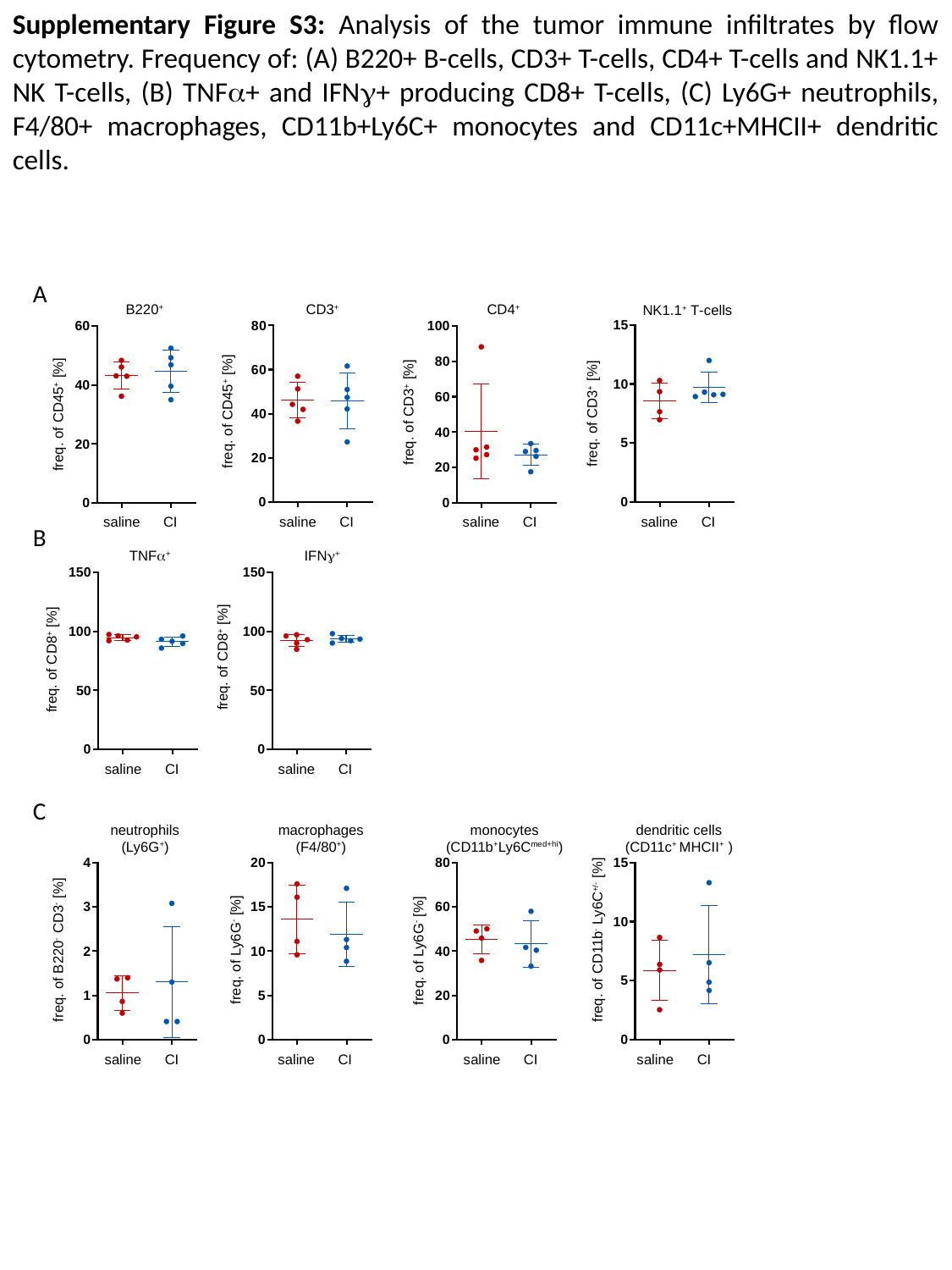

Supplementary Figure S3: Analysis of the tumor immune infiltrates by flow cytometry. Frequency of: (A) B220+ B-cells, CD3+ T-cells, CD4+ T-cells and NK1.1+ NK T-cells, (B) TNFα+ and IFNγ+ producing CD8+ T-cells, (C) Ly6G+ neutrophils, F4/80+ macrophages, CD11b+Ly6C+ monocytes and CD11c+MHCII+ dendritic cells.
A
B220+
CD3+
CD4+
NK1.1+ T-cells
freq. of CD45+ [%]
freq. of CD3+ [%]
freq. of CD3+ [%]
freq. of CD45+ [%]
saline
CI
saline
CI
saline
CI
saline
CI
B
TNFα+
IFNγ+
freq. of CD8+ [%]
freq. of CD8+ [%]
saline
CI
saline
CI
C
neutrophils (Ly6G+)
macrophages (F4/80+)
monocytes (CD11b+Ly6Cmed+hi)
dendritic cells
(CD11c+ MHCII+ )
freq. of CD11b- Ly6C+/- [%]
freq. of B220- CD3- [%]
freq. of Ly6G- [%]
freq. of Ly6G- [%]
saline
CI
saline
CI
saline
CI
saline
CI

## Slide 5
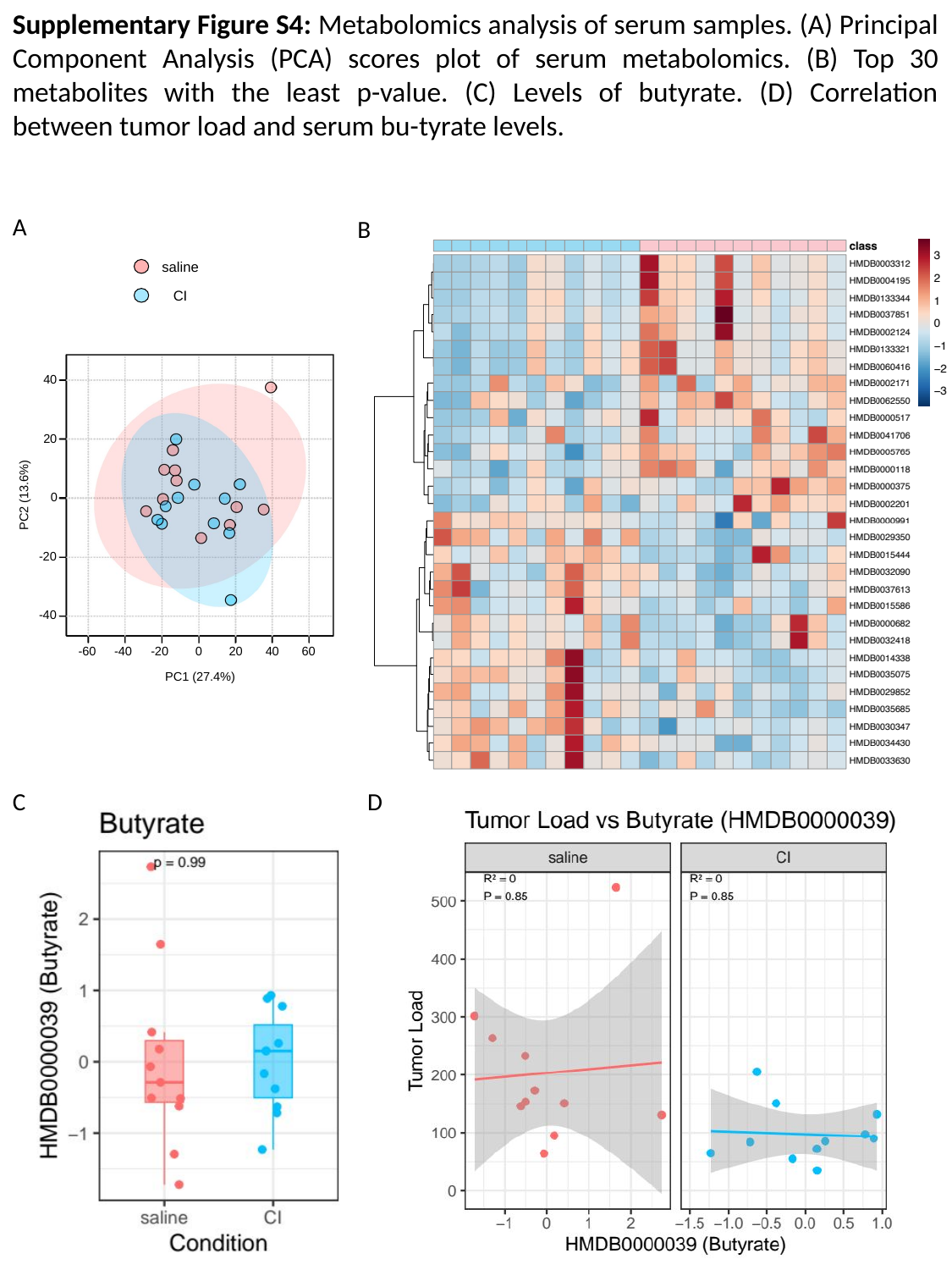

Supplementary Figure S4: Metabolomics analysis of serum samples. (A) Principal Component Analysis (PCA) scores plot of serum metabolomics. (B) Top 30 metabolites with the least p-value. (C) Levels of butyrate. (D) Correlation between tumor load and serum bu-tyrate levels.
A
B
saline
CI
40
20
PC2 (13.6%)
0
-20
-40
-60
-40
-20
0
20
40
60
PC1 (27.4%)
C
D
